# High Risk of Under-Grading and -Staging in Prostate Cancer Patients Eligible for Active Surveillance

**DOI:** 10.1371/journal.pone.0115537

**Published:** 2015-02-06

**Authors:** Isabel Heidegger, Viktor Skradski, Eberhard Steiner, Helmut Klocker, Renate Pichler, Andreas Pircher, Wolfgang Horninger, Jasmin Bektic

**Affiliations:** 1 Medical University of Innsbruck, Department of Urology, Innsbruck, Austria; 2 Medical University of Innsbruck, Department of Haematology and Oncology, Innsbruck, Austria; Gentofte University Hospital, DENMARK

## Abstract

**Background:**

Active surveillance (AS) is increasingly offered to patients with low risk prostate cancer. The present study was conducted to evaluate the risk of tumor under-grading and -staging for AS eligibility. Moreover, we analyzed possible biomarkers for predicting more unfavorable final tumor histology.

**Methods:**

197 patients who underwent radical prostatectomy (RPE) but would have met the EAU (European Association of Urology) criteria for AS (PSA<10 ng/ml, biopsy GS ≤6, ≤2 cancer-positive biopsy cores with ≤50% of tumor in any core and clinical stage ≤T2a) were included in the study. These AS inclusion parameters were correlated to the final histology of the RPE specimens. The impact of preoperative PSA level (low PSA ≤4 ng/ml vs. intermediate PSA of >4–10 ng/ml), PSA density (<15 vs. ≥ 15 ng/ml) and the number of positive biopsy cores (1 vs. 2 positive cores) on predicting upgrading and final adverse histology of the RPE specimens was analyzed in uni- and multivariate analyses. Moreover, clinical courses of undergraded patients were assessed.

**Results:**

In our patient cohort 41.1% were found under-graded in the biopsy (final histology 40.1% GS7, 1% GS8). Preoperative PSA levels, PSA density or the number of positive cores were not predictive for worse final pathological findings including GS >6, extraprostatic extension and positive resection margin (R1) or correlated significantly with up-grading and/or extraprostatic extension in a multivariate model. Only R1 resections were predictable by combining intermediate PSA levels with two positive biopsy cores (p = 0.004). Sub-analyses showed that the number of biopsy cores (10 vs. 15 biopsy cores) had no influence on above mentioned results on predicting biopsy undergrading. Clinical courses of patients showed that 19.9% of patients had a biochemical relapse after RPE, among all of them were undergraded in the initial biopsy.

**Conclusion:**

In summary, this study shows that a multitude of patients fulfilling the criteria for AS are under-diagnosed. The use of preoperative PSA levels, PSA density and the number of positive cores were not predictable for undergrading in the present patient collective.

## Introduction

Prostate cancer (PCa) is the most common cancer and the second cause of cancer death among men in European countries [1]. In general, PCa is a highly heterogeneous disease, ranging from slow-growing indolent tumors to rapidly progressing highly aggressive carcinomas associated with significant morbidity and mortality. Therefore, early detection of PCa by measuring prostate specific antigen (PSA) values at regular intervals in peripheral blood is important to identify men with aggressive cancers at early stage [2].

Generally, organ confined PCa can be cured either by radical prostatectomy (RPE) or primary radiation therapy. However, in the recent years active surveillance (AS) is promoted as an approach with the aim to reduce overtreatment of patients with low risk PCa. AS implicates the decision not to treat the patient immediately, but to make a close follow up and to treat patients at pre-defined thresholds that classify progression such as short PSA doubling time and deteriorating Gleason Score (GS) on repeat biopsy [3]. According to the guidelines of the European Society of Urology (EAU) AS can be offered to patients with the lowest risk of cancer progression implicating clinical stage T1-2a, PSA < 10 ng/mL, biopsy GS≤ 6 (at least 10 biopsy cores), ≤2 positive biopsies as well as minimal biopsy core involvement (≤ 50% cancer per biopsy) [4].

In the recent years there is a growing body of evidence to support the safety of AS instead of immediate treatment for low risk cancer. Surgery implicates side effects like urinary incontinence, loss of fertility and potency as well as possible intra-or postoperative complications. Also the use of radiation therapy has been associated with side effects including urinary incontinence, radiation-cystitis or secondary cancerous malignancies.

The aim of AS of early prostate cancer is to individualize therapy by selecting for curative therapy only patients with significant cancers.

Based on early data, demonstrating that men with differentiated PCa have a 20-year PCa-specific survival rate of 80–90% without any therapy [5,6], AS is an option in the management of low-risk PCa patients that aims to reduce the risk of over-treatment. Recent data from the PRIAS study showed a disease-specific survival rate of 100% on AS in a median follow-up of 1.6 years [7]. Another prospective study found that only 5/453 patients developed metastatic disease and died from PCa providing support for an AS approach in favorable low risk PCa [8].

Several studies investigated the impact of several biomarkers for predicting disease progression in patients undergoing AS. An overview about selected studies addressing this issue is shown in Table 1. In addition to these other molecular markers like TMSS-ERG fusions or the use of MRI fusion techniques have been reported [4].

**Table 1:** Overview about clinical studies on biomarkers predicting undergrading; + positive association,—negative association.

Number of patients, % undergrading	PSA	PSA density	Number of positive biopsy cores	Others	Reference (Pubmed ID)
197, 41.1%	-	-	-		Own study
67, 44.8%	+	Not assessed	Not assessed		22921697
274, 34.1%	Not assessed	+	Not assessed	Age	23820059
241, 23%	+	Not assessed	Not assessed		21419438
376, 32.7%	+	+	+	Free PSA	20304433
135, 30%	Not assessed	+	Not assessed		21167525
60, not assessed.	Not assessed	+	Not assessed	KI67	18762814
2449, 28%	+	Not assessed	+	PSA doubling time	23159452
4500, 50% (all GS 6 tumors)	+	+	Not assessed	Age, >4mm disease on core, palpable tumor	24071481
757, 21.5%	+	+	+		21704447

The aim of this study was to evaluate the accuracy of staging and grading at the time of biopsy in patients who met the inclusion criteria for AS according to the EAU guidelines [4]. Moreover, we evaluated PSA and the number of positive cores as prediction markers as well as the impact of PSA density (< 0.15 vs ≥ 0.15 ng/ml) for unfavorable final tumor histology, including higher GS, extraprostatic extension and positive resection margin.

## Patients and Methods

### Ethics Statement

The study has been approved by the ethics committee of the Medical University of Innsbruck (study number AM3174, number of the positive votum of the Ethics: UN3174, AM 3174). Written informed consent was given by participants for their clinical records to be used in this study.

A retrospective analysis of 197 patients who underwent RPE due to biopsy verified GS6 PCa between 1996 and 2009 has been performed. Age adjusted PSA levels in combination with percent-free PSA (fPSA) of less than 18% as well as digital rectal examinations were used as criteria for transrectal ultrasound-guided prostate biopsy [9] (Table 2). To diagnose PCa from 1995 to March 2000, 10 systematic transrectal ultrasound (TRUS) guided prostate biopsy cores were taken in a standard spatial distribution by experienced urologists; from April 2000 to 2009 additional five doppler-enhanced targeted biopsies cores were taken on the basis of age-specific PSA reference ranges by an experienced uroradiologist. When more than one core was taken from the same area of the prostate only one core counted as a positive punch. Grey scale TRUS was done using a Combison 530MT unit (Kretztechnik, Zipf, Austria) fitted with a biplanar probe operating at a frequency of 10 MHz. No template based perineal biopsies or MRI-fusion biopsies were performed.

**Table 2:** Age adjusted biopsy criteria.

Age range (years)	PSA range (ng/ml)
40–49	0–1.25
50–59	0–1.75
60–69	0–2.25
70–75	0–3.25

Each biopsy core was analyzed by an experienced pathologist at Department of Pathology of Medical University Innsbruck.

All patients met the EAU criteria for AS [4] and underwent either open retropubic (1996–2009) or robot-assisted laparascopic (2001–2009) RPE without lymph node extirpation. Biopsy and RPE specimens were analyzed by an experienced uro-pathologist at the Medical University of Innsbruck, Department of Pathology, Innsbruck, Austria.

The correlation of GS 6 in needle biopsy and corresponding RPE specimen was analyzed. Patients were stratified by preoperative PSA levels into a low (≤4 ng/ml) and an intermediate (>4–10 ng/ml) PSA group. Moreover, patients were stratified according to the number of positive biopsies as well according to PSA density <0.15 or ≥0.15 ng/ml. PSA density was calculated as following: total serum PSA / prostate volume. Additionally, the incidence of extraprostatic extension (≥pT3a) and positive surgical margins (R1) was analyzed.

### Statistics

Statistical calculations were performed using SPSS for Windows (SPSS, Chicago, Illinois, USA). Chi Quadrat test and Fisher’s test were used for evaluation of differences between groups. Moreover, multivariate analyses and logistic regressions were calculated using SPSS. P-values below 0.05 were considered significant (* p< 0.05).

## Results

We investigated a cohort of 197 patients who underwent RPE between 1995–2009 and met the EAU criteria for AS including PSA <10 ng/ml, biopsy GS 6 with ≤2 cancer-positive cores and ≤50% of any core involved with tumor, clinical stage ≤T2a [4]. All patients were Caucasians and the median age at biopsy was 60.2 years (range 40.9 to 75.2 years). The median preoperative PSA level was 4.10 ng/ml (range 1.87 ng/ml to 9.9 ng/ml). Patients were stratified according to preoperative PSA levels into a low (≤4 ng/ml, 46.2% of patients) and an intermediate (>4–10 ng/ml, 53.8% of patients) PSA group, according to the number of positive biopsies into a 1 and a 2 positive cores group (60. 4% and 39.6%, respectively) as well as according to PSA density (<0.15 vs ≥ 0.15 ng/ml) (Table 3).

**Table 3:** Patient characteristics, Statistics: descriptive.

PSA category (ng/ml)	n (%) total
PSA ≤4	91 (46.2%)
PSA 4–10	106 (53.8%)
Positive cores category	n (%)
1 core	119 (60.4%)
2 cores	78 (39.6%)
PSA density (ng/ml)	n (%)
<0.15	128 (65.0%)
≥0.15	50 (25.4%)
Missing	19 (9.6%)

A needle biopsy grade of GS 6 was upgraded in the final RPE histology in 81 patients (41.1%). Final grade GS 7 was found in 79 (40.1%), final grade GS 8 in 2 (1%) patients (Table 3). Concerning GS 7 tumors, 72/79 tumors were 3+4 tumors, while 7/79 were diagnosed as 4+3 tumors (Table 4).

**Table 4:** Final GS grade and pathological tumor stage of RPE specimen Statistics: descriptive.

Gleason Score RPE	n (%)
Gleason Score 5	34 (17.3%)
Gleason Score 6	82 (41.6%)
Gleason Score 7 total	79 (40.1%)
Gleason Score 7 3+4	72 (36.6%)
Gleason Score 7 4+3	7 (3.5%)
Gleason Score 8	2 (1%)
pT stadium RPE	n (%)
pT2a	45 (22.8%)
pT2b	11 (5.6%)
pT2c	128 (65%)
pT3a	11 (5.6%)
pT4	2 (1%)
Surgical margin	n (%)
≤pT2c R0	157 (79.7%)
≤pT2c R1	27 (13.7%)
≥pT3a R0	6 (3.0%)
≤pT3a R1	7 (3.6%)

We wondered if time from biopsy to RPE has an influence on undergrading. Thus, we compared the average time from biopsy to RPE between undergraded and not-undergraded patients. The mean time from biopsy to RPE was 92.5 days in the not-undergraded group (median: 80.5, SD 79.16), while it was 82.7 days in the undergraded group of patients (median: 74.5, SD 59.74). Statistical analyses revealed that time from biopsy to RPE was not a significant factor for undergrading (p = 0.356).

As many patients eligible to AS were upgraded in the final histology, we aimed to identify predictive factors for upgrading. First, we evaluated the *number of positive biopsy cores* and found no significant correlation between number of positive cores and under-grading in the biopsy (p = 0.568). In line with these findings, the number of positive biopsy cores was not able to predict extraprostatic extension (≤ pT2c vs. ≥pT3a: p = 0.208) or positive surgical margin (p = 0.033).

Next we evaluated the impact of *preoperative PSA level* on final histology upgrading and found no significant impact (p = 0.116). Also extra-prostatic extension (p = 0.248) and positive surgical margins (p = 0.031) in the RPE specimen were not predictable by preoperative PSA levels.

Moreover, we investigated the impact of *PSA density* as prediction marker for adverse pathological findings. In line with the number of positive cores and preoperative PSA levels PSA density was not able to predict upgrading (p = 0.718) or extraprostatic extension (p = 0.186). However, the risk for positive surgical margins was significantly predictable by PSA density ≥ 15 ng/ml (p = 0.000).

Employing multivariate analysis we tested the *combination of preoperative PSA level*, *the number of positive biopsy cores* and *PSA density* for predicting an unfavorable final pathology. In line with univariate analyses most combinations of parameters were not able to predict final worse pathological findings including higher GS and extra-prostatic extension (Table 5). Interestingly, only the combinations of intermediate PSA level + only one positive biopsy core as well as PSA density ≥ 15 ng/ml + intermediate PSA + one positive biopsy core were associated with significant higher number of pT3a tumors (Table 5). Concerning the prediction of both, pT3a tumors and undergrading only the combination PSA density ≥ 15 ng/ml + intermediate PSA + one positive biopsy core was predictive (Table 5).

Positive resection margins were predictable by four different combinations of parameters with the highest impact by combining PSA density ≥15 ng/ml/ intermediate PSA and two biopsy cores (p = 0.000) (Table 5). However, logistic regression confirmed no improvement of prediction of PCa aggressiveness by preoperative PSA value, by the number of positive biopsy cores, by PSA density or by combination of these parameters (Table 6).

**Table 5:** Multivariate model; PSA levels (ng/ml) the number of positive biopsy cores and PSA density (ng/ml) for predicting worse final pathological findings; Statistics: *Chi-Quadrat Pearson; **Fisher test.

Category	Undergrading	pT2c versus pT3a	R1	pT3a+undergrading
PSA ≤4+1 core	0.272* 0.274**	0.265* 0.266**	0.044* (sig) 0.045** (sig)	0.418*0.419**
PSA >4–10+1 core	0.638* 0.639**	0.016* (sig) 0.015** (sig)	0.776* 0.777**	0.090* 0.091**
PSA ≤4+2 cores	0.448* 0.449**	0.853* 0.854**	0.665* 0.666**	0.934* 0.934**
PSA >4–10+2 cores	0.174* 0.175**	0.190* 0.191**	0.004* (sig) 0.004** (sig)	0.278 0.279**
Density <0.15+PSA≤4+1 core	0.300* 0.302**	0.450* 0.452**	0.038* (sig) 0.039** (sig)	0.558* 0.559**
Density <0.15+PSA >4–10+1 core	0.464* 0.465**	0.504* 0.506**	0.272* 0.274	0.579* 0.580**
Density <0.15+PSA ≤4+2 cores	0.795* 0.796**	0.903* 0.904**	0.906* 0.906**	0.784 0.785
Density <0.15+PSA >4–10+ 2 cores	0.141* 0.142**	0.861* 0.861**	0.139* 0.141**	0.943* 0.944**
Density ≥0.15+PSA ≤4+1 core	0.821* 0.821**	0.715* 0.716**	0.222* 0.223**	0.729* 0.729**
Density ≥0.15+PSA >4–10+1 core	0.985* 0.985**	0.003* (sig) 0.003** (sig)	0.792* 0.792**	0.019* (sig)0.020** (sig)
Density ≥0.15+PSA ≤4+ 2 cores	0.392* 0.393**	0.797* 0.797**	0.645* 0.646**	0.807* 0.807**
Density ≥0.15+PSA >4–10+2 cores	0.690* 0.691**	0.210* 0.212**	0.000* (sig) 0.000** (sig)	0.234* 0.234**

**Table 6:** Logistic regression for predicting PCa aggressiveness.

Variables for testing	AUC
1 vs. 2 positive biopsy cores	0.520
PSA ≤4 vs. PSA 4–10	0.557
Density <0.15 vs. ≥0.15	0.512
PSA ≤4 + 1 positive biopsy core	0.464
PSA 4–10 + 1 positive biopsy core	0.479
PSA ≤4 + 2 positive biopsy cores	0.516
PSA 4–10 + 2 positive biopsy cores	0.541
Density <0.15 + PSA ≤4 + 1 core	0.465
Density <0.15 + PSA >4–10 + 1 core	0.521
Density <0.15 + PSA ≤4 + 2 cores	0.493
Density <0.15 + PSA >4–10+ 2 cores	0.534
Density ≥0.15 + PSA ≤4 + 1 core	0.502
Density ≥0.15 + PSA >4–10 + 1 core	0.501
Density ≥0.15 + PSA ≤4 + 2 cores	0.495
Density ≥0.15 + PSA >4–10+ 2 cores	0.490

Statistics: Logistic regression (AUC), PSA (ng/ml), PSA density (ng/ml).

Moreover, we performed sub-analyses of differences in biopsy techniques 10 cores vs. 15 cores. Thereby we found that undergrading was higher in the group of 15 biopsy cores (44.5%) vs. 19.2% in the group of 10 cores. However, it has to be considered that the number of patients within the groups highly differs (n = 26 vs. n = 171) (Table 7). Final GS of RPE specimens (10 vs. 15 cores) are shown in Table 8. Multivariate analyses revealed that in both groups neither PSA levels, the number of positive cores nor PSA density (in all combinations) were able to predict undergrading (Table 9–10). Also AUC curves did not differ significantly between 10 and 15 biopsy cores (Table 11).

**Table 7:** Patient characteristics sub-analyzed according to biopsy cores (10 cores versus 15 cores), Statistics: descriptive.

PSA category (ng/ml)	n (%) 10 cores	n (%)15 cores
Total	26 (13.2%)	171 (86.8%)
PSA ≤4	13 (50%)	78 (45.6%)
PSA 4–10	13(50%)	93 (54.4%)
Positive cores category	n (%)	n (%)
1 core	15(57.7%)	104 (60.8%)
2 cores	11 (42.3%)	67 (39.2%)
PSA density (ng/ml)	n (%)	n (%)
<0.15	13 (50%)	115(67.3%)
≥0.15	3 (11.5%)	47 (27.5%)
Missing	10 (38.5%)	9 (5.2%)

**Table 8:** Final GS grade and pathological tumor stage of RPE specimen sub-analyzed according to biopsy cores (10 cores versus 15 cores), Statistics: descriptive.

Gleason Score RPE	n (%)10 cores	n (%)15 cores
Total	26 (13.2%)	171 (86.8%)
Gleason Score 5	7 (26.9.%)	27 (15.8%)
Gleason Score 6	14 (53.8.%)	68 (39.8%)
Gleason Score 7 total	5 (19.2%)	74 (43.3%)
Gleason Score 7 3+4	1 (3.8%)	68 (39.8%)
Gleason Score 7 4+3	1 (3.8%)	6 (3.5%)
Gleason Score 8	0 (0%)	2 (1.2%)
pT stadium RPE	n (%)	n (%)
pT2a	5 (19.2%)	40 (23.4%)
pT2b	6 (23.1%)	5 (2.9%)
pT2c	13 (50%)	115 (67.3%)
pT3a	1 (3.8%)	10 (5.8%)
pT4	1 (3.8%)	1 (0.6%)
Surgical margin	n (%)	n (%)
≤pT2c R0	21 (80.8%)	136 (79%)
≤pT2c R1	3 (11.5%)	24 (14%)
≥pT3a R0	1 (3.8%)	5 (2.9%)
≤pT3a R1	1 (3.8%)	6 (3.5%)

**Table 9:** Multivariate model.

Category	Undergrading	pT2c versus pT3a	R1	pT3a+ undergrading
PSA ≤4+ 1 core	0.689	0.372	0.925	0.536
PSA >4–10+1 core	0.619	0.540	0.036 (sig)	0.497
PSA ≤4+2 cores	0.856	0.347	0.234	0.630
PSA >4–10+2 cores	0.961	0.473	0.289	0.691
Density <0.15+PSA ≤4+1 core	0.182	0.551	0.712	0.551
Density <0.15+PSA >4–10+ 1 core	0.712	0.620	0.356	0.420
Density <0.15+PSA ≤4+ 2 cores	0.712	0.032 (sig)	0.356	0.032 (sig)
Density <0.15+PSA >4–10+2 cores	0.712	0.620	0.356	0.620
Density ≥0.15+PSA ≤4+1 core	No data	No data	No data	No data
Density ≥0.15+PSA >4–10+1 core	0.383	0.696	0.002 (sig)	0.696
Density ≥0.15+PSA≤4+2 cores	No data	No data	No data	No data
Density ≥0.15+PSA >4–10+2 cores	0.551	0.790	0.620	0.790

PSA levels (ng/ml) the number of positive biopsy cores and PSA density (ng/ml) for predicting worse final pathological findings in the population of 10 biopsy cores, Statistics: Chi-Quadrat Pearson test.

**Table 10:** Multivariate model.

Category	Undergrading	pT2c versus pT3a	R1	pT3a+ undergrading
PSA ≤4+1 core	0.276	0.405	0.035 (sig)	0.508
PSA >4–10+1core	0.741	0.018 (sig)	0.285	0.046 (sig)
PSA ≤4+2 cores	0.548	0.500	0.962	0.575
PSA >4–10+2 cores	0.179	0.262	0.001 (sig)	0.320
Density <0.15+PSA ≤4+1 core	0.448	0.543	0.023 (sig)	0.673
Density <0.15+PSA >4–10+1 core	0.489	0.591	0.853	0.678
Density <0.15+PSA ≤4+2 cores	0.734	0.559	0.398	0.645
Density <0.15+PSA >4–10 +2 cores	0.113	0.989	0.219	0.898
Density ≥0.15+PSA ≤4+1+1 core	0.859	0.715	0.218	0.730
Density ≥0.15+PSA >4–10+1 core	0.817	0.001 (sig)	0.647	0.010 (sig)
Density ≥0.15+PSA ≤4+2cores	0.376	0.797	0.502	0.808
Density ≥ 0.15+ PSA >4–10+2 cores	0.713	0.221	0.000 (sig)	0.247

PSA levels (ng/ml) the number of positive biopsy cores and PSA density (ng/ml) for predicting worse final pathological findings in the population of 15 biopsy cores, Statistics: Chi-Quadrat Pearson test

**Table 11:** Logistic regression for predicting PCa aggressiveness sub-analyzed 10 versus 15 biopsy cores; PSA (ng/ml), PSA density (ng/ml), Statistics: Logistic regression (AUC).

Variables for testing	10 cores AUC	15 cores AUC
1 vs. 2 positive biopsy cores	0.542	0.512
PSA ≤4 vs. PSA 4–10	0.625	0.541
Density <0.15 vs. ≥0.15	0.458	0.520
PSA ≤4 + 1 positive biopsy core	0.333	0.474
PSA 4–10 + 1 positive biopsy core	0.625	0.514
PSA ≤4 + 2 positive biopsy cores	0.542	0.484
PSA 4–10 + 2 positive biopsy cores	0.500	0.528
Density <0.15 + PSA ≤4 + 1 core	0.333	0.473
Density <0.15 + PSA >4–10 + 1 core	0.542	0.520
Density <0.15 + PSA ≤4 + 2 cores	0.542	0.490
Density <0.15 + PSA >4–10+ 2 cores	0.542	0.537
Density ≥0.15 + PSA ≤4 + 1 core	0.500	0.502
Density ≥0.15 + PSA >4–10 + 1 core	0.583	0.493
Density ≥0.15 + PSA ≤4 + 2 cores	0.500	0.495
Density ≥0.15 + PSA >4–10+ 2 cores	0.458	0.490

**Fig 1:**
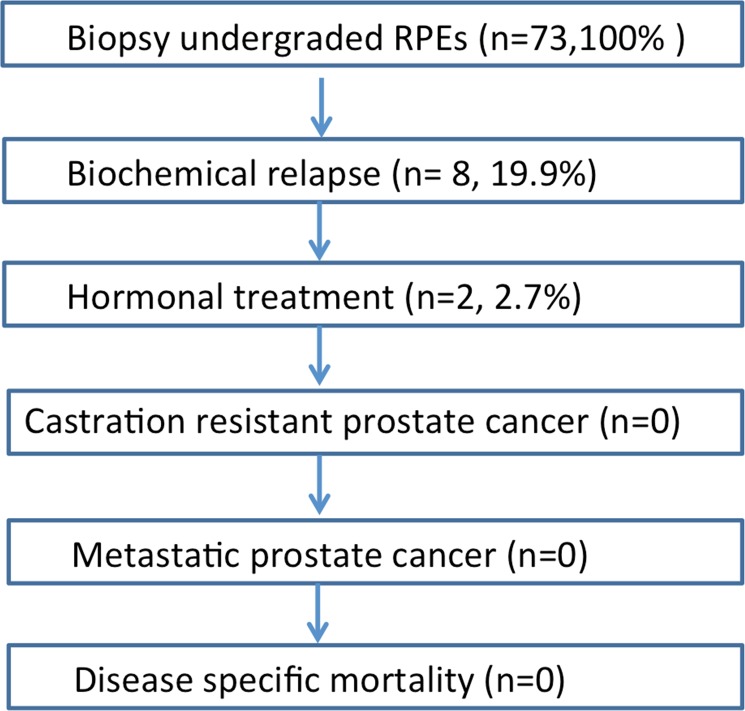
Follow up of biopsy-undergraded patients treated with radical prostatectomy, n = 81, 7 patients´ follow up missing→ n = 73, 100%.

At last we analyzed those patients who were biopsy undergraded. Thereby we found that in our patient collective the incidence of biochemical relapse treated by radiation therapy (72Gy) after RPE was 19.2% (n = 8), while only 2.7% (n = 2) were treated hormonally (Fig. 1). Interestingly, all 8 patients, who had a biochemical relapse after RPE, were undergraded in the initial biopsy. Among these, 7 patients had a GS7 in the RPE specimen and 1 patient showed a GS8 in the RPE specimen. Both patients with hormonal treatment were undergraded in the biopsy.

## Discussion

Many prostate tumors, especially in elderly men, are estimated to have a protracted natural history. However, the majority of men diagnosed with PCa undergo aggressive treatment regardless of risk and changing landscape of PCa. Consequently, a considerable part of PCa is over-treated. For these patients AS could represent an adequate treatment option [10,11].

The present study was undertaken to evaluate the risk of under-grading and -staging when applying the criteria for eligibility for AS. Evaluating 197 patients we found that 41.1% of patients were under-graded. Thus, a large number of patients initially eligible for AS would not meet the AS criteria based on final histology. This finding is in line with a small study involving 67 patients that also found a significant under-estimation of GS in the biopsy compared to the corresponding RPE specimen [12]. Several other studies showed that the needle biopsy GS correlates only in about 60 to 75% accurately with the RPE GS [13,14]. Recently, our group described that 52.4% of patients were under-graded in the biopsy [15].

The GS determined in the prostate needle biopsy is an essential component in the algorithm for treatment selection. An under-grading rate of 41.1% found in the present study underscores the risk and consequence of undergrading at biopsy in a group of patients that would have been eligible for conservative management. Thus it seems important to inform patients about the risk of under-grading when considering AS in low risk PCa.

Several studies have been conducted or are ongoing to define predictive factors or methods to improve precise staging of PCa: Fleshner et al for example used repeat biopsies as strategy to improve the reliability of needle biopsy grading in patients with well differentiated PCa (n = 165) [16]. However, repeated biopsies harbor an increased risk of perioperative complications including bleeding complication or febrile prostatitis. Recently, Ehdaie et al for example found that in men with PCa on AS the number of previous prostate biopsies is associated with a significant risk of infectious complications (n = 403) [17]. Han et al described low preoperative PSA as predictor for insignificant PCa (n = 67) [12]. In contrast to this finding, our study indicates that preoperative PSA levels cannot predict biopsy under-grading. Moreover, our study clearly shows that also the number of positive biopsy cores as well as the combination of both factors is not able to predict worse final pathological findings. Again, this finding is in contrast to a large study including 757 patients that found the number of positive cores (two vs. one positive core) at initial biopsy to be predictive for reclassification of PCa one year later [18]. Possible explanations for the different findings in our study may be the fact, that most studies that described PSA as predictor for undergrading involved a larger patient collective. Therefore one could speculate that the statistical power of this study is too low for make a definitive statement.

Additionally we investigated in this study the impact of PSA density as a possible marker for predicting unfavorable pathological findings. Several studies support the inclusion of PSA density, rather than PSA, into the risk stratification system for patients seeking less invasive treatment for PCa. Oh et al for example found that PSA density may be a significantly more accurate preoperative predictor of upgrading than PSA (n = 505) [19]. Moreover, a recently published population study including 4500 men showed that PSA density ≥0.15 ng/ml is a predictor for adverse pathology (upgrading to GS 7 or higher, or up staging to pT3 or greater) [20]. Our findings are in contrast to these findings where we did not find PSA density ≥0.15 ng/ml as predictor for undergrading or extraprostatic extension. Only positive surgical margins were predictable by PSA density ≥0.15 ng/ml.

For a majority of patients with low risk PCa, AS offers the benefit of personalized medicine, avoiding treatment and possible negative effects on quality-of-life. Several studies addressed the quality-of-life issues in men managed in the AS regime compared to those with RPE. A recent study evaluated the prevalence of depression, anxiety and distress among AS and RPE patients and found no significant difference [21]. However, radical treatment often implicates significant side effects which often have an enormous impact on patients´ quality-of-life that would be avoided by selecting AS. A recent longitudinal study for example including 374 men showed that men in the RPE group consistently reported more leakage, impaired erection and libido, and fewer obstructive voiding symptoms compared to the watchful waiting group [22].

Clinical courses of patients clearly showed that all patients who had biochemical relapse after RPE were undergraded in the biopsy. This finding indicates that undergrading in patients stratified for AS lead to increased PCa progression. These data illustrate again, that AS regime has to be selected very carefully especially in young men.

Strengths of the present study are the homogenous population due to uniformly applied inclusion criteria and the large patient collective. Another important aspect of our findings is that PCa diagnosis was made through extended biopsy protocols (10–15 biopsies), which is known to improve diagnosis and reduce sampling errors.

A limitation of the study is that we analyzed a highly selected screening population from the Tyrol study. Moreover, it has also to be considered, that the potential under-estimation of GS and tumor extent may also result from sampling bias, variation in biopsy numbers and inter-observer variability among pathologists for grading of PCa. Although all biopsies were taken according to an internal scheme, biopsies were performed by multiple individuals which could be an additional confounding factor in the biopsy outcomes.

## Conclusion

AS for favorable risk PCa is an approach that may reduce over-treatment of clinically insignificant PCa. However, our results show that 41.1% of patients treated by RPE at our institution who would have met the inclusion criteria for AS, had a worse final histology and therefore would not have been destined for followed-up in an AS strategy. Neither preoperative PSA levels nor the number of positive biopsy cores or PSA density were predicting adverse final pathological findings. Only positive surgical margins were predictable by PSA density ≥0.15 ng/ml. Moreover, we found that undergraded patients have a higher risk of PCa progression.

These findings should be considered in the decision process regarding the treatment of patients with localized PCa especially for those with a life expectancy of >10 years. Therefore it is important to inform the patient about the possibility of under-grading in the prostate biopsy. An unresolved problem remains the lack of accurate markers for definition of significant or insignificant disease and an increased confidence towards conservative treatment of PCa.
